# Effect of Visual Exposure versus Obstruction upon Patient's Quantitative and Qualitative Stress Parameters Changes during Minor Oral Surgery

**DOI:** 10.1055/s-0042-1757212

**Published:** 2022-10-11

**Authors:** Pram Kumar Subramaniam, Basma Ezzat Mustafa Al-Ahmad, Nazih Shaban Mustafa, Natasha Athirah Mohd Izhan, Nur Farah Izzati Ahmad Shukor

**Affiliations:** 1 Department of Oral Maxillofacial Surgery and Oral Diagnosis, Faculty of Dentistry, International Islamic University Malaysia, Kuantan, Pahang, Malaysia; 2 Department of Fundamental Dental Medical Sciences, Kulliyyah of Dentistry, International Islamic University Malaysia, Kuantan, Pahang, Malaysia; 3 O'Hara Orthodontics Specialist Dental Clinic, Kuantan, Pahang, Malaysia; 4 Klinik Pergigian Mentakab, Pahang, Malaysia

**Keywords:** stress parameters, visual, minor oral surgery, draping

## Abstract

**Objectives** Dental anxiety is ultimately related to the fear of pain, more evidently seen in surgical dental procedures. This study aimed at comparing the stress parameter differences between patients undergoing minor oral surgery (MOS) with their eyes covered (closed) versus uncovered (open) at our center.

**Materials and Methods** Twenty-three MOS patients were draped with eyes covered, while another 23 MOS patients were draped with eyes uncovered. Stress parameters such as systolic and diastolic blood pressures (DBP), mean arterial pressure (MAP), heart rate, random blood glucose, and Spielberger State-Trait Anxiety Inventory (STAI)-6 questionnaire score were recorded accordingly in the different intervals; then statistically analyzed later.

**Results** Closed eyes patients had significantly lower mean DBP and MAP (73.91 ± 6.80/88.94 ± 6.88 mm Hg) as compared with open eyes patients intraoperatively. Though significant only in the postoperative phase, the closed eyes group had a relatively lower mean heart pulse rate than the open eyes group in all surgical intervals. Postoperatively, closed eyes patients had lower mean blood glucose level as compared with open eyes group. STAI mean score revealed a higher psychological stress for closed eyes patients versus open eyes patients.

**Conclusion** Closed eyes patients displayed lower quantifiable physiological stress level as compared with patients undergoing MOS draped with eyes uncovered. However, in qualitative psychological context, closed eyes draped MOS patients responded poorly as compared with opened eyes draped patients under similar surgical stress.

## Introduction

Dentophobia or dental anxiety refers to the abnormal panic sensation which is induced during dental procedures, 1 commonly sensitized by past traumatic experience of pain. Some of the highest rated dental anxiety dental procedures include local anesthesia injection, bone drilling, and tooth removal. During a surgical procedure, the body manifests stress responses incorporating a wide range of endocrine, immunological, and hematological effects. 2 3 4 Psychosomatic changes can be induced by dental treatment that becomes uncomfortable for the patient, resulting in anxiety inducing autonomic heart rate (HR) changes. 5 Similarly, hypertension may be aggravated by dental treatment even in a normal patient. A previously cited article has reported the contribution of sympathetic nervous response under stress to the regulation of blood glucose changes. 6 The state of anxiety can be corresponded to level of fear or anxiety and classified as response time and intensity, or in other tools that could reflect upon the person's disposition and predictor status of postoperative symptoms. 7

Anxiety or stress in relation to oral surgery can be regulated with external stimuli as reported by several studies. It is concluded that musical intervention helps reduce the patient's anxiety during surgical removal of the impacted mandibular third molar. 8 In 2015, Bakar reported that hearing at Holy Quran recitals helps Muslim patients in the intensive care unit to modify their HR and is recommended as an intervention for psychospiritual comfort for Muslims. 9 Likewise, musical intervention during the stressful surgical phase can be seen through alteration in the sympathetic nervous activity.

The stress also presented a role of modulation in immune response due to their reduction in defense capacity. As a consequence of this mechanism, the organism turns more susceptible to developing psychosomatic and inflammatory diseases. 10 11

Controlling the stress indirectly should have a great effect on the healing process of the wound after minor oral surgery (MOS) to highlight the postoperative complications of patients having uncovered (opened eyes) versus covered (closed eyes).

In our study, we aim to observe the changes of stress related to MOS changes with modification of the patient's visual sensory input by comparing the stress parameter of patients having their eyes uncovered (open eyes) versus eyes covered (closed eyes) during the preoperative, intraoperative interval, and postoperative phase.

In fact, when this study concept is narrowed within the realm of third molar surgery, then the results shown here are of unique originality and pave the path toward new knowledge discovery. Hence, this study is expected to be a reference for future research of similar intent. Surgical stress and anxiety observation following visual stimuli modification during third molar surgery is a subject matter that has yet to be expounded, and the results will definitely fill the knowledge void.

## Materials and Methods

### Study Protocol

This prospective randomized clinical controlled trial study was approved by the Ethical Review Board of International Islamic University Malaysia, Kuantan Campus, Malaysia (Ref: IIUM/305/14/11/2/IREC 558) and performed according to standard Helsinki guidelines.

In the test group, the patients undergoing MOS (mandibular third molar surgery) had surgical draping that did not cover their eyes. Their eyes were protected by fitting clear goggles. The control group patients underwent MOS with their eyes covered according to draping in the standard manner. All the patients involved were informed regarding the study protocol and had signed a written consent.

### Sample Size Calculation

To calculate sample size, G*Power software program was used. 12 Using data from previous systematic review study, the systolic blood pressure (SBP) reduction was taken as the main outcome with a mean difference value of 2.6 mm Hg and a standard deviation of 1.285 noted between test and control groups. 13 Expected effect size was tabulated at 2.0057, while α value was set at 0.05 along with the power of study at 95% while keeping the ratio of distribution at 1:1. With these conditions, eight patients per group were calculated as the minimum sample size.

### Patients

A total of 50 patients planned for surgical removal of mandibular third molar at the Kulliyyah of Dentistry, International Islamic University Malaysia (IIUM) student polyclinic were selected as sample for this study. The patient selected as subjects in this research had to abide by the inclusion criteria such as general healthiness, aged between 18 and 40 years, indicated for surgical removal of mandibular third molar, 14 and should have taken their meal at least 2 hours before commencement of the procedure. However, any patients who had undergone previous MOS or dentoalveolar surgery less than a year ago, or having medically compromised issues or substance abuse or drug addiction or consuming antipsychotic or antidepressant drugs were excluded from this study. Similarly, patients suffering from acute oral inflammation were also discarded from this study.

The patients were randomly divided into two groups using list randomizing software ( www.randomizer.org ). The randomized list was created by a surgeon not directly involved with the procedures or data analysis. Four patients were dropped out due to ineligibility according to preset criterion. Hence, only 23 patients for each group were allotted in the end.

### Variables and Measurement

SBP and diastolic blood pressure (DBP) and HR were measured with an electronic digital device (Medisana Blood Pressure Monitor MTP) and recorded. Only one machine was used throughout this study. The device has a maximum error tolerance for pressure of ± 3 mm Hg and pulse rate (PR) of ± 5% in its reading and conforms to the European Norm standard EN 606011-1-2. The blood pressure and PR readings were taken preoperatively, intraoperatively, and finally, at postoperative stage. Mean arterial pressure (MAP) for each interval was calculated using the formula MAP = DBP + 1/3 (SBP − DBP). 15

Next, the random blood glucose (RBG) levels were determined preoperatively and postoperatively by drawing blood via finger pinprick. The electronic strips containing drawn blood were fed into electronic glucometer (TRUEresult Blood Glucose Meter) providing blood glucose level in millimole per liter. Only a single machine was used throughout this study and produce results with mean absolute relative error value of 4.2%, meeting the ISO accuracy requirements of EN ISO 15197:2003.

Patient's psychological anxiety status was recorded with the Spielberger State-Trait Anxiety Inventory (STAI) questionnaire form. This assessment tool was preferred over others as it has been used extensively and has been considered a valid tool in several cited studies. 16 17 For this research, a modified type of STAI was used, the six-item questionnaire. 18 Considering the use in the Malaysian populace, the questionnaire was made into a bilingual version, with English and Malay version under the assistance of a certified linguist. The translated form was checked for validity and later approved by the IIUM Research Ethics Committee (IREC). The STAI-6 questionnaire has a reliability of 90% and predictability of 89% 19 saving time from answering the full questionnaire. The range of score is between 6 (minimum) and 24 (maximum), higher score indicating an increase in psychological stress. 20 This questionnaire was administered preoperative and postoperatively.

### Procedures

The test group patients had full visual input of their surroundings as their eyes were not blocked by surgical drapes, while the control group had standard surgical draping applied covering patient's visual sight as well. Proper information and details of the study protocol were explained to the patients, including the objectives, confidentiality of the data, and withdrawal rights. Written consent was obtained from each patient to ensure there was no violation of rights. 5 Consequently, preoperative reading of blood pressure, PR, and pricking for blood glucose level were done and recorded. The patients then answered a six-question STAI questionnaire preoperatively.

**Fig. 1:**
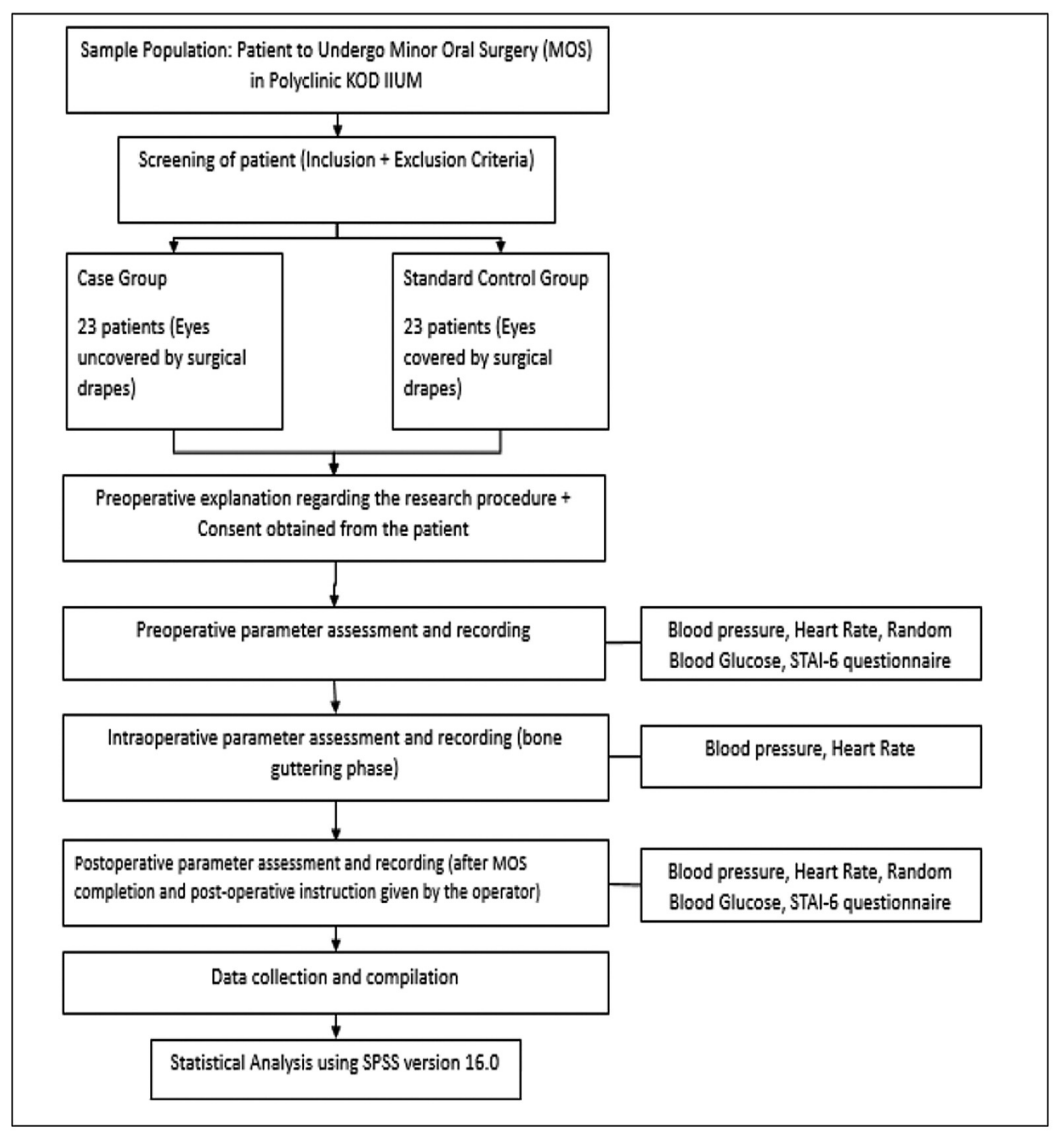
Flowchart of research methodology.

Each mandibular third molar surgery was done according to the highest standard of infection control and standard surgical protocol used at the Faculty of Dentistry, IIUM. All procedures were conducted in the presence of the same surgeon with 7 years' experience. The intraoperative readings of blood pressure and PR were taken during the bone removal stage. Upon completion of surgery, the patient again answered another set of STAI questionnaire once their blood pressure, PR, and blood glucose record were repeated ( Fig. 1 ).

### Clinical Biostatistics

Data analyses were made by using IBM SPSS Statistic for Windows (Version 26.0) software. 21 All data were presented as mean ± standard deviation.

## Results

The study was conducted over a period of 4 months and was concluded with 23 patients in each group. The demographic distribution of patients based on gender, age group, and racial profile appears to be statistically similar between control and test groups ( Table 1 ).

**Table 1:** Patient demographic distribution between test and control groups

		Open eyes (test)	Closed eyes (control)
Race	Malay	21	22
	Chinese	2	1
Gender	Male	6	6
	Female	17	17
Age group	18–20	2	4
	21–25	18	16
	25–30	3	3

In general, the mean SBP and DBP between open eyes (test) and closed eyes (control) group at various intervals were not significantly different from each other as shown in Table 2 . An obvious difference between test and control groups' SBP and DBP was evident during intraoperative phase. Test group showed a mean SBP of 123.39 ± 16.78 mm Hg, higher than control group (119.00 ± 11.12 mm Hg) intraoperatively. Statistically, mean DBP of test group (79.04 ± 10.24 mm Hg) is significantly higher than control group (73.91 ± 6.80 mm Hg) in intraoperative phase. Interestingly, control group displayed an increasing pattern of SBP mean value from preoperative to postoperative interval, while test group exhibits a spike of SBP mean value from preoperative to intraoperative phase and plateaus in postoperative phase.

**Table 2:** Comparison of mean SBP and DBP between test and control groups

Surgical interval	SBP (mm Hg) Mean ± standard deviation		Sig.	DBP (mm Hg) Mean ± standard deviation		Sig.
	Open eyes (test)	Closed eyes (control)		Open eyes (test)	Closed eyes (control)	
Preoperative	116.17 ± 17.45	117.74 ± 10.99	0.088	76.04 ± 13.61	75.70 ± 11.86	0.705
Intraoperative	123.39 ± 16.78	119.00 ± 11.12	0.097	79.04 ± 10.24	73.91 ± 6.80	0.048 a
Postoperative	123.26 ± 16.11	124.17 ± 12.61	0.597	86.74 ± 13.56	85.35 ± 14.20	0.976

Abbreviations: DBP, diastolic blood pressure; SBP, systolic blood pressure.

a *p* -Value less than 0.05.

Next, the mean value of MAP was statistically significant during the intraoperative phase between open and closed eyes groups with the *p* -value of 0.013. MAP mean of test group (93.83 ± 11.84 mm Hg) was greater as compared with control group (88.94 ± 6.88 mm Hg) intraoperatively. MAP mean values between the groups in the other intervals were close to each other as shown in Table 3 . Incidentally, both groups exhibited a rising MAP value from preoperative to postoperative stage. Also, in Table 3 , the mean PR values between open eyes (test) and closed eyes (control) groups were not significantly different with the exception of postoperative phase. It is worth to noted that both subject groups had high PR value at the intraoperative phase before dropping down.

**Table 3:** Comparisons of MAP and PR mean value between open eyes (test) group and closed eyes (control) group

Intervals	MAP (mm Hg) Mean ± standard deviation		Sig.	PR (bpm) Mean ± standard deviation		Sig.
	Open eyes (test)	Closed eyes (control)		Open eyes (test)	Closed eyes (control)	
Preoperative	89.42 ± 14.01	89.71 ± 11.06	0.358	86 ± 15.0	78 ± 10.2	0.303
Intraoperative	93.83 ± 11.84	88.94 ± 6.88	0.013 a	85 ± 11.0	81 ± 12.8	0.643
Postoperative	98.91 ± 13.63	98.29 ± 13.14	0.905	80 ± 8.4	79 ± 14.5	0.022 a

Abbreviations: MAP, mean arterial pressure; PR, pulse rate.

a *p* -Value less than 0.05.

**Fig. 2:**
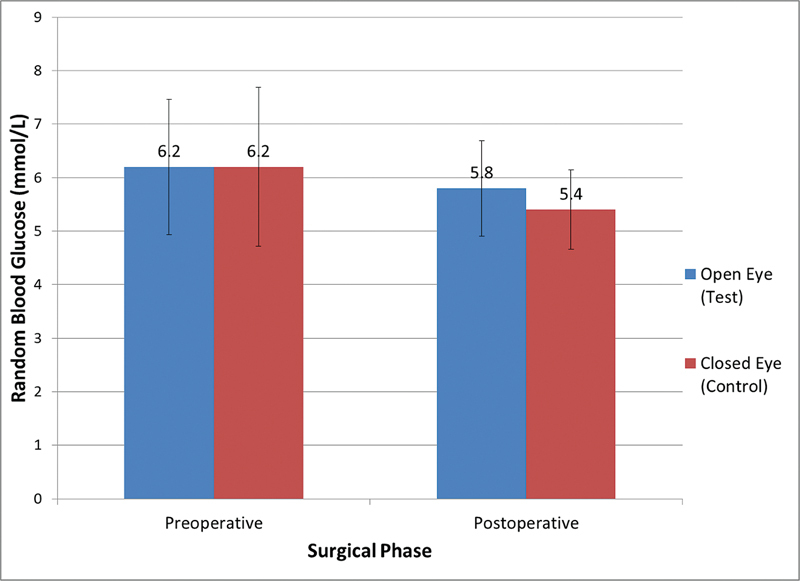
Comparison of preoperative and postoperative mean random blood glucose levels between open eyes (test) and closed eyes (control) groups.

RBG mean value was noted to be fairly same for open eyes (6.2 ± 1.26 mmol/L) and closed eyes (6.2 ± 1.48 mmol/L) groups (see Fig. 2 ). However, open eyes subjects recorded a higher RBG mean (5.8 ± 0.74 mmol/L), while the closed eyes group reported mean RBG of 5.4 ± 0.74 mmol/L. However, this reading of mean RBG between open eyes (test) and closed eyes (control) groups was not statistically significant as indicated by the *p* -value being more than 0.05.

**Fig. 3:**
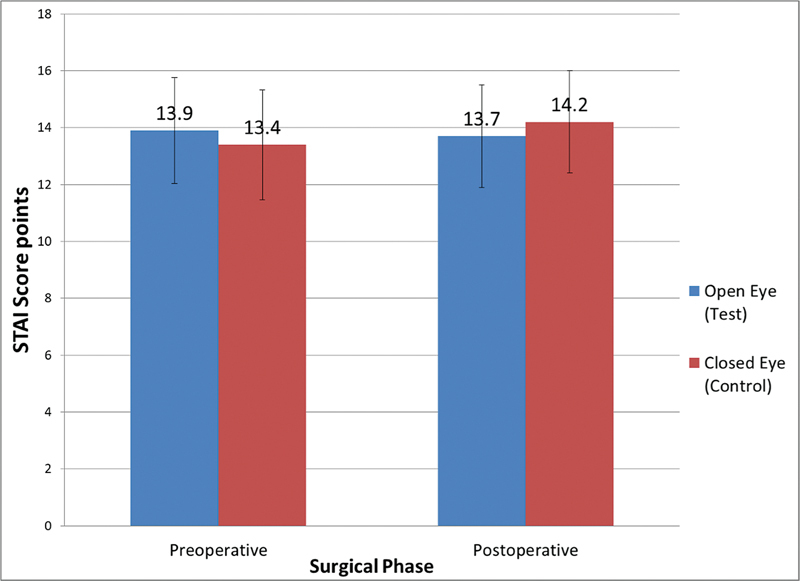
Comparison of preoperative and postoperative mean STAI scores between open eyes (test) and closed eyes (control) group. STAI, Spielberger State-Trait Anxiety Inventory.

Fig. 3 shows the bar chart comparing mean value of STAI score among closed eyes and open eyes groups at preoperative and postoperative intervals. Both mean score sets were not statistically significant due to the *p* -value more than 0.05. The pattern of the STAI score in closed eyes group shows a slightly increase in score, 14.2 during the postoperative, while preoperative shows 13.4 mean score. Open eyes group however shows a slight reduction in the STAI score, reduction of 13.9 in preoperative to 13.7 postoperatively. These differences are very menial and do not express significant changes. What is interesting though is that while the open eyes group's STAI score did not change preoperative to postoperatively, the closed eyes group's score increased by 0.8.

## Discussion

With the scope of search within the context of this study, the author has noted that the study of anxiety or stress assessment between subject eyes covered or eyes open during a local aesthetic procedure or similar research is few.

In this present study, the mean SBP of patients in the test group versus the control group was not significantly different. This is in agreement with the findings by Luque-Ribas et al which compared the level for third molar surgery patients with video eyeglasses versus eyes covered with drapes 14 where the mean SBP did not show any significant variation between surgical phases. Additionally, in the present study, the mean DBP of the test group was significantly higher than control group which was contrasting in the Luque-Ribas et al's study. 14 The blood pressure response toward surgical procedure can be influenced by many factors such as the effect of vasoconstrictor within local anesthesia 22 as well as the sympathetic activation of endogenous epinephrine. The higher blood pressure reading in the open eyes group is postulated to coincide with the rate of sympathetic activation as part of the flight-or-fight response caused by visual stimuli. 23

MAP was used to measure the adequacy of blood perfusion into vital tissues and organs. Although anxiety response is expected to be the highest during bone guttering phase, it is noteworthy that closed eyes group had lower MAP. This is not reported by a review report from Pittman and Kridli 24 that states how MAP is not affected by musical intervention and does not change between test and control groups.

Our article could infer that open eyes patients would have found the surgical experience unnerving during the aforementioned phase leading to an elevated MAP following excretion of corticotrophin-releasing factor, increasing plasma catecholamine, elevated HR, and vasopressin. 25

In the present study, we have noted that with the exception of the postoperative phase, there are no significant differences in PR between test and control subjects. However, it is noted that both groups showed high mean PR in the intraoperative phase, which is supported by Luque-Ribas et al who reported that noted intraoperative phase of drilling and incision showed the highest mean HR. 14 Similarly, a study by Tarazona-Álvarez et al reported consistently stating that the HR peaks at the intraoperative phase namely during incision and bone ostectomy or drilling phase in third molar surgery. 26 These hemodynamic changes are agreed to be manifestation of the stress response, namely, in anticipation of impending dental treatment which is further influenced by painful stimuli, action of catecholamines in local anesthetics, and also physical stress. 27

Next, we have noticed that mean blood glucose between subjects of the test and control groups were not significantly changed in both preoperative and postoperative phases of the procedure. This finding was consistent with a study by Kaviani et al that studied the average glucose level was similar at different interval of implant procedures for subject under influence of midazolam or propofol. 28 It implies how the stress response is not being affected despite different drug used.

STAI score represents the psychological response and is a qualitative measure of the patient's stress or anxiety. The increase in STAI score indicates an increase in the psychological stress. 20 The control and test groups in this study were recognized to have moderate anxiety according to STAI categorization: mild (scores 6–11), moderate, 12 13 14 15 16 17 and severe anxiety. 18 19 20 21 22 23 24 Result noted in this study suggests that anxiety level is consistent between test and control groups both preoperatively and postoperatively. This finding is similar to finding seen in report by Luque-Ribas et al who reported no significant changes between experiment and control groups. 14 Another study by Choi et al compared anxiety level of patients who were presented with audio–visual slide presentation of treatment information versus written information that noted lower anxiety level following third molar surgery for the audio–visual group. 29 This finding was not consistent with the present study. Studies such as reported by Kazancioglu et al that noted subjects who are exposed to audio–visual stimuli regarding the surgical procedure are likely to increase anxiety and pain postoperatively. 30 It is surmised that such audio–visual stimuli detailing surgical procedure namely incision, tooth grinding, and bone removal are likely to incite more fear and anxiety, and this is lesser if the audio–visual stimuli is obliterated as in the case of closed eyes drape subjects. This feature was visible in the hemodynamic aspect but not in the anxiety level in current study.

## Conclusion

Overall, this study has shown that the draping method to include the covering of eyes could reduce surgical-related stress intraoperatively. Stress management is important since human internal stress mechanisms can delay healing and reduce the poor surgical prognosis as mentioned in our literature reviews. Practicing dental surgeons performing MOS should employ the method of surgical draping with the eyes covered not only as upholding standards for aseptic surgery but also to minimize stress to patients.
